# Multifractal detrended fluctuation analysis of human gait diseases

**DOI:** 10.3389/fphys.2013.00274

**Published:** 2013-10-07

**Authors:** Srimonti Dutta, Dipak Ghosh, Sucharita Chatterjee

**Affiliations:** ^1^Department of Physics, Behala College, University of CalcuttaKolkata, India; ^2^UGC Emeritus Fellow, Physics Department, Jadavpur UniversityKolkata, India; ^3^Department of Physics, Bangabasi College, University of CalcuttaKolkata, India

**Keywords:** non-stationary time series, gait disease, multifractal, monofractal, degree of multifractality, degree of correlation

## Abstract

In this paper multifractal detrended fluctuation analysis (MFDFA) is used to study the human gait time series for normal and diseased sets. It is observed that long range correlation is primarily responsible for the origin of multifractality. The study reveals that the degree of multifractality is more for normal set compared to diseased set. However, the method fails to distinguish between the two diseased sets.

## Introduction

Neurodegenerative disease is an umbrella term for a range of conditions which primarily affect the neurons in the human brain and the spinal cord. Neurons are the building blocks of the nervous system which includes the brain and the spinal cord. Neurons normally do not reproduce or replace themselves, so when they become damaged or die out, they cannot be replaced by the body. Many neurodegenerative diseases including Parkinson's disease (PD), Huntington's disease (HD), amyotrophic lateral sclerosis (ALS), or Alzheimer's disease (AD) occur as a result of neuro-degeneration, which is a term for progressive loss of structure or function of neurons, including the death of neurons.

Degenerative nerve diseases cause worsening of many of body activities, balance, movement, talking, breathing, heart function and even mental functioning (dementia). Many of the diseases are genetic while certain medical conditions such as alcoholism, a tremor or a stroke can cause other types. These diseases can be serious or even life threatening. It depends on their type. Most of these diseases are incurable. The goal of the treatment is usually to improve symptoms, relieve pain and increase mobility.

PD is a degenerative disorder of the central nervous system. In PD the dopamine generating cells in the substantia nigra, a region of the midbrain die out. Dopamine sends signals to that part of the brain that controls movement. Early in the course of the disease, the most obvious symptoms are movement related. These include shaking, rigidity, slowness of movement and difficulty walking and gait. In time, PD affects muscles all through the body and leads to problems like trouble speaking, trouble swallowing, or constipation. Later cognitive and behavioral problems may arise, with dementia commonly occurring in the advanced stages of the disease. PD usually begins around the age of 60, but it even starts earlier. It is more common in men than in women. There is no cure for PD. A variety of medicines sometimes help symptoms dramatically.

HD is a neurodegenerative genetic disorder that affects muscle coordination and leads to cognitive decline and psychiatric problems. It typically becomes noticeable in mid-adult life. It is an inherited disease that causes certain nerve cells in the brain to waste away.

The disease is caused by an autosomal dominant mutation in either of an individual's two copies of a gene called Huntington, which means any child of an affected person typically has a 50% chance of inheriting the disease. People are born with the defective gene, but symptoms usually don't appear until middle-age. The earliest symptoms are often subtle problems with mood or cognition. Early symptoms of HD may include uncontrolled movement, clumsiness or balance problems. A lack of coordination and an unsteady gait often follows. As the disease advances, uncoordinated, jerky body movements become more apparent along with a decline in mental abilities and behavioral and psychiatric problems. Later this disease can take away the ability to walk, talk or swallow. Mental abilities generally decline into dementia; some people even stop recognizing family members. There is no cure for HD and full-time care is required in the later stages of the disease. Medicines can help manage some symptoms, but cannot slow down or stop the disease.

In this paper a multifractal detrended fluctuation analysis (MFDFA) of human gait time series for diseased and controlled set is performed. Now it may arise in the mind of the reader that what is a fractal and what is its relation with human gait?

The term “fractal” was first coined by Mathematician Benoît Mandelbrot (1982) in 1975. Mandelbrot based it on the Latin adjective “fractus” meaning “broken” or “fractured.” Fractal geometry mathematically characterizes systems that are basically irregular at all scales. A fractal structure has the property that if a small portion of the system is magnified, it shows the same complexity as the entire system. Fractals can be classified into two categories: monofractals and multifractals. Monofractals are those, whose scaling properties are the same in different regions of the systems and Multifractals are complicated self-similar objects consisting of differently weighted fractals with different non-integer dimensions. As a result a multifractal system is a generalization of a fractal system in which a single scaling exponent is not enough to describe its dynamics; instead a continuous spectrum of exponents (the so called singularity spectrum) is needed.

Hausdorff et al. (2001) have demonstrated strong connection between human walking and random walk. Though walking appears to be a periodic regular process the gait pattern reveals small fluctuations even under stationary conditions. They employed detrended fluctuation analysis (DFA) to show that the fluctuations in stride interval exhibit long range correlations.

In recent past, the DFA has become a very useful technique to determine the fractal scaling properties and long-range correlations in noisy, non-stationary time-series. It has been widely applied to diverse fields such as DNA sequences, heart rate dynamics, neuron spiking, human gait, and economic time-series and also to weather related and earthquake signals (Ossadnik et al., 1994; Peng et al., 1994; Buldyrev et al., 1995, 1998; Blesic et al., 1999; Liu et al., 1999; Bunde et al., 2000; Talkner and Weber, 2000; Ashkenazy et al., 2001). But DFA has its own limitations. Many geophysical signals as well as medical patterns do not exhibit monofractal scaling behavior, which can be accounted for by a single scaling exponent (Hu et al., 2001; Kantelhardt et al., 2001), therefore different scaling exponents are required for different parts of the series (Chen et al., 2002). Consequently a multifractal analysis should be applied. The MFDFA was first conceived by Kantelhardt et al. (2002) as a generalization of the standard DFA. MFDFA has been applied successfully to study multifractal scaling behavior of various non-stationary time series (Kantelhardt et al., 2003; Telesca et al., 2004, 2005; Sadegh Movahed et al., 2006; Lan et al., 2008; Niu et al., 2008; Shang et al., 2008; Yuan et al., 2009).

Fractal properties of the human neuromuscular system has been observed in ECG, electroencephalogram (EEG) recordings of brain waves as well as in recordings of human movement, such as walking gait, running gait, standing posture, and eye movements (Goldberger et al., 2002a; Scafetta et al., 2003, 2007, 2009; West and Scafetta, 2003; Zhou et al., 2007; Van Orden et al., 2009; Dutta, 2010a,b; Coey et al., 2012). Fractal properties are an emergent property of the system dynamics and that certain pathology can disrupt its dynamics resulting in the alternation of its fractal properties. There are several methods that have been used to study human gait for normal and diseased set (Hausdorff et al., 1996, 1997, 2000, 2001; Goldberger et al., 2002a,b; Scafetta et al., 2003, 2007, 2009; West and Scafetta, 2003; Van Orden et al., 2009). DFA, a scaling technique, introduced by Hausdorff et al., to study the dynamics of human gait under different walking rates, has been proved to be effective (Hausdorff et al., 1996). It was observed that scaling was degraded in certain diseased states such as PD and HD in their later work (Hausdorff et al., 1996, 1997, 2000, 2001; Goldberger et al., 2002b). Scafetta et al. (2009) have observed that human stride interval is complex time series that is characterized by particular symmetries including fractal and multifractal properties using the SCPG technique. The randomness of the fluctuations is found to be higher in elderly or cases with neurodegenerative diseases. In this respect it would be interesting to extend the detrending technique used by Hausdorff et al. (2001) designed for monofractal series to multifractal formalism MFDFA. Long range correlation properties of the gait series have been given a lot of emphasis in all the previous studies. Here we have also tried to find a quantitative estimation of degree of multifractality and study its variation among the diseased and control set.

## Description of data

Neurodegenerative diseases often affect gait and mobility. The stride-interval is a measure of the gait rhythm and is typically defined as the time from heel strike to next heel strike of the same foot. The stride interval, the time between consecutive heel strikes of the same foot has been seen to fluctuate from one strike to the next in a complex fashion (Yamasaki et al., 1984; Pailhous and Bonnard, 1992; Hausdorff et al., 1995).

In order to investigate the fractal properties of the human gait in case of normal persons (Control Group) and patients with PD and HDs, we studied the databases of human gait from website www.physionet.org. The records in the neuro-degenerative disease are from patients with PD, HD and records from healthy subjects (Control Group) has been included as the comparison group.

## Method of analysis

We have performed a multifractal analysis of the stride-time fluctuations of human gait in three cases, (i) healthy persons (Control Group), (ii) persons with PD, and (iii) persons with HD following the prescription of Kantelhardt et al. (2002). The important steps involved in this method of analysis are mentioned here:

**Step1:** Computing the average

Let us suppose *x*(*i*) for *i* = 1 … *N*, be a non-stationary time series of length *N*. The mean of the above series is given by

(1) $$x_{\text{ave}}=\frac{1}{N}\sum_{i\;=\;1}^{N}x(i)$$

**Step 2:** Computing the integrated time series

(2) $$Y(i)\equiv \overset{i}{\underset{k=1}{\sum }}\left[x(k)-x_{\text{ave}}\right]\text{ for }i=1\ldots N$$

**Step 3:** Dividing the integrated time series to *N*_*s*_ non-overlapping bins (where *N*_*s*_ = int(*N*/*s*) and *s* is the length of the bin) and computing the fluctuation function. Since *N* is not a multiple of *s*, so in order to include this part of the series the entire process is repeated starting from the opposite end. Thus, 2*N*_*s*_ bins are obtained and for each bin we perform least square fit of the series and then determine the variance

$$F^{2}(s,\nu )=\frac{1}{s}\sum_{i=1}^{s}{\left\{Y[(\nu -1)s+i]-y_{\nu }(i)\right\}}^{2}$$

for each bin ν, ν = 1, … *N*_*s*_ and

$$F^{2}(s,\nu )=\frac{1}{s}\sum_{i=1}^{s}{\left\{Y[N-(\nu -N_{s})s+i]-y_{\nu }(i)\right\}}^{2}$$

for ν = *N*_*s*_ + 1, …, 2*N*_*s*_ where *y*_ν_(*i*) is the least square fitted value in the bin ν. We have adopted MFDFA 1 which uses a least square linear fit.

**Step4:** Computing fluctuation function

The *q*th order fluctuation function *F*_*q*_(*s*) is obtained after averaging over 2*N*_*s*_ bins.

(3) $$F_{q}(s)={\left\{\frac{1}{2N_{s}}\sum_{\nu =1}^{2N_{s}}{\left[F^{2}(s,\nu )\right]}^{\frac{q}{2}}\right\}}^{\frac{1}{q}}$$

where *q* is an index which can take all possible values except zero because in that case the factor 1/*q* blows up. *F*_*q*_ cannot be obtained by the normal averaging procedure; instead a logarithmic averaging procedure is applied

(4) $$F_{0}(s)\equiv \exp\;\left\{\frac{1}{4N_{s}}\sum_{\nu =1}^{2N_{s}}\ln[F^{2}(s,\nu )]\right\}\sim s^{h(0)}$$

**Step 5:** The procedure is repeated by varying the value of *s*.*F*_*q*_(*s*) increases with increase in value of *s*. If the series is long range power correlated, then *F*_*q*_(*s*) will show power law behavior

$$F_{q}(s)\propto s^{h(q)}$$

If such a scaling exists ln *F*_*q*_(*s*) will depend linearly on ln *s*, with *h*(*q*) as the slope. In general the exponent *h*(*q*) depends on *q*. For stationary time series *h*(2) is identical with the Hurst exponent H. *h*(*q*) is said to be the generalized Hurst exponent. A monofractal time series is characterized by unique *h*(*q*) for all values of *q*.

The generalized Hurst exponent *h*(*q*) of MF-DFA is related to the classical scaling exponent τ(*q*) by the relation

(5) τ(q)=qh(q)−1

A monofractal series with long range correlation is characterized by linearly dependent *q* order exponent τ(*q*) with a single Hurst exponent *H*. Multifractal signal have multiple Hurst exponent and τ(*q*) depends non-linearly on *q* (Ashkenazy et al., 2003a).

The singularity spectrum *f*(α) is related to *h*(*q*) by

(6) $$\alpha =h(q)+qh^{\prime }(q)$$

(7) f(α)=q[α−h(q)]+1

where α is the singularity strength and *f*(α) specifies the dimension of subset series that is characterized by α. The multifractal spectrum is capable of providing information about relative importance of various fractal exponents in the series e.g., the width of the spectrum denotes range of exponents. A quantitative characterization of the spectra may be obtained by least square fitting it to a quadratic function (Shimizu et al., 2002) around the position of maximum α_0_,

(8) $$f(\alpha )=A{\left(\alpha -\alpha_{0}\right)}^{2}+B(\alpha -\alpha_{0})+C$$

where *C* is an additive constant *C* = *f*(α_0_) = 1. *B* indicates the asymmetry of the spectrum. It is zero for a symmetric spectrum. The width of the spectrum can be obtained by extrapolating the fitted curve to zero. Width *W* is defined as

(9) $$W=\alpha_{1}-\alpha_{2}$$

with *f*(α_1_) = *f*(α_2_) = 0. It has been proposed by some groups (Ashkenazy et al., 2003b) that the width of the multifractal spectra is a measure of degree of multifractality. For a monofractal series, *h*(*q*) is independent of *q*. Hence from relation (6) and (7) it follows that the width of the spectrum will be zero for a monofractal series. The more the width, the more multifractal is the spectrum.

The origin of multifractality in a time-series can be determined. Two basic sources of multifractality in the time-series are:

- Multifractality due to broad probability density function for the values of the time-series.
- Multifractality due to different long-range correlations of the small and large fluctuations.

The origin of the multifractality can be ascertained by analyzing the corresponding randomly shuffled series. In the shuffling procedure, the values are put into random order and hence all correlations are destroyed. Hence, if the multifractality is due to long-range correlations, then the shuffled series exhibits a non-fractal scaling. On the other hand, if the original *h*(*q*) dependence does not change, i.e., *h*(*q*) = *h*_shuffled_(*q*), then the multifractality is due to the broad probability density, which is not affected in the shuffling procedure. If both kinds of multifractality are present in a given series, the shuffled series will show weaker multifractality than the original series.

The autocorrelation exponent γ can be estimated from the relation given below: (Kantelhardt et al., 2002; Sadegh and Hermanis, 2008)

(10) γ=2−2h(q=2)

For uncorrelated or short-range correlated data, *h*(2) is expected to have a value 0.5 while a value greater than 0.5 is expected for long-range correlations. Therefore, for uncorrelated data, γ has a value 1 and the lower the value the more correlated is the data.

## Results and discussion

The normal gait time-series is highly inhomogeneous and non-stationary and fluctuates about the mean value in an irregular and complex manner. Gait rhythm of the (i) healthy persons (Control Group), (ii) persons with PD, and (iii) persons with HD have been studied using the MFDFA method of analysis of a non-stationary time series.

**Figure 1:**
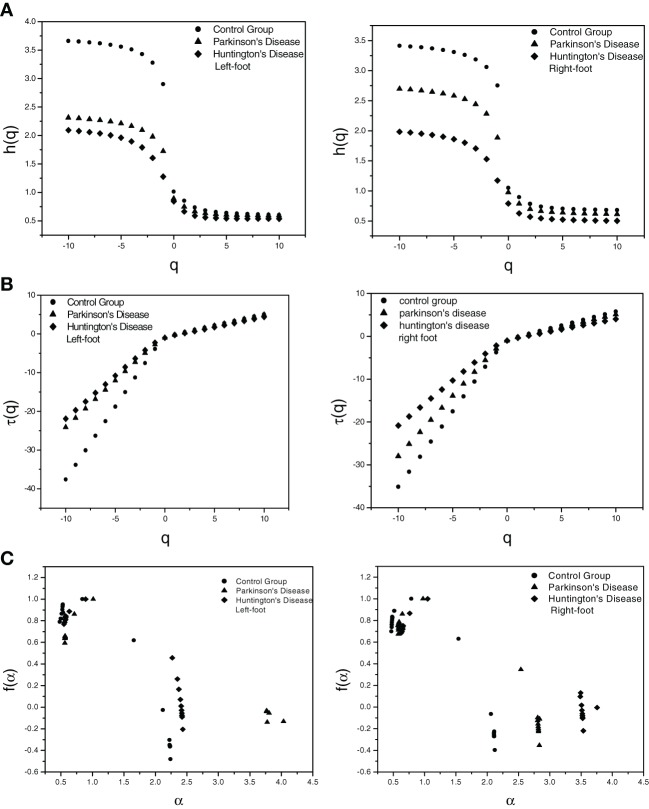
**Plot of (A) Generalized Hurst exponent *h*(*q*) vs. order *q* (B) Classical Scaling exponent (Equation 5) τ(*q*) vs. order *q* and (C) Dimension of subset series (Equation 6) *f*(α) vs. Singularity strength (Equation 7) α for one set each of three different cases for left-foot and for right-foot**.

The data for each case was transformed to obtain the integrated signal. The integrated time-series was then divided into *N*_*s*_ non-overlapping bins. The value of *s* was chosen in the range 5 to *N*/5 in steps of 1 (*N* = 1200). The *q*th order fluctuation function *F*_*q*_(*s*) for *q* = −10 to +10 in steps of 1 was obtained. The linear dependence of ln *F*_*q*_(*s*) on ln *s* for different orders of *q* for the three groups, namely (i) healthy persons (Control Group), (ii) persons with PD, and (iii) persons with HD, was observed which indicates a scaling behavior. The slope of the linear fit to ln *F*_*q*_(*s*) vs. ln *s* plot gives the values of *h*(*q*). One representative figure for variation of *h*(*q*) with *q* for each of the three cases is provided in Figure 1A. The variation of *h*(*q*) with *q* indicates a multifractal behavior It is evident from Figure 1A that the values of *h*(*q*) decreases with increasing *q*.

The values of the classical scaling exponents τ(*q*) were also calculated using relation (5) for each *q* for all the three cases. From Figure 1B depicting the variation of τ(*q*) with *q*, it is evident that τ(*q*) has a non-linear dependence on *q* suggesting a multifractal nature of the series, while for monofractal series τ(*q*) would depend linearly on *q*. The variation of *h*(*q*) with *q* and the non-linear dependence of τ(*q*) on *q* reflects the existence of multifractality in human gait in all the three cases.

The degree of multifractality in each case can be determined quantitatively. We have calculated α and *f*(α) using relation (6) and (7). The multifractal spectrum, the singularity spectrum [*f*(α) vs. α] is shown in Figure 1C. The multifractal spectra were fitted to the quadratic equation (8).

The width *W* of the spectrum is a measure of the degree of multifractality. The mean values of width *W* of the multifractal spectra listed in Table 1 shows that the width of multifractal spectrum is greater in case of the healthy subject (Control Group) than those for patients with PD and HD which suggests that the degree of multifractality is more in case healthy subjects than those with neurodegenerative diseases.

**Table 1:** **Mean values, Variance of multifractal width *W* (Equation 9), and ANOVA parameters *F* and *p* values for all three groups**.

	**Values of *W***		***F***	***p***
	**Average**	**Variance**		
Control left-foot	3.7	2.1	8.79	0.002
Parkinson's left-foot	2.2	0.1		
Huntington's left-foot	2.15	0.03		
Control right-foot	3.8	1.2	6.25	0.008
Parkinson's right-foot	2.8	1.1		
Huntington's right-foot	2.3	0.3		

**Figure 2:**
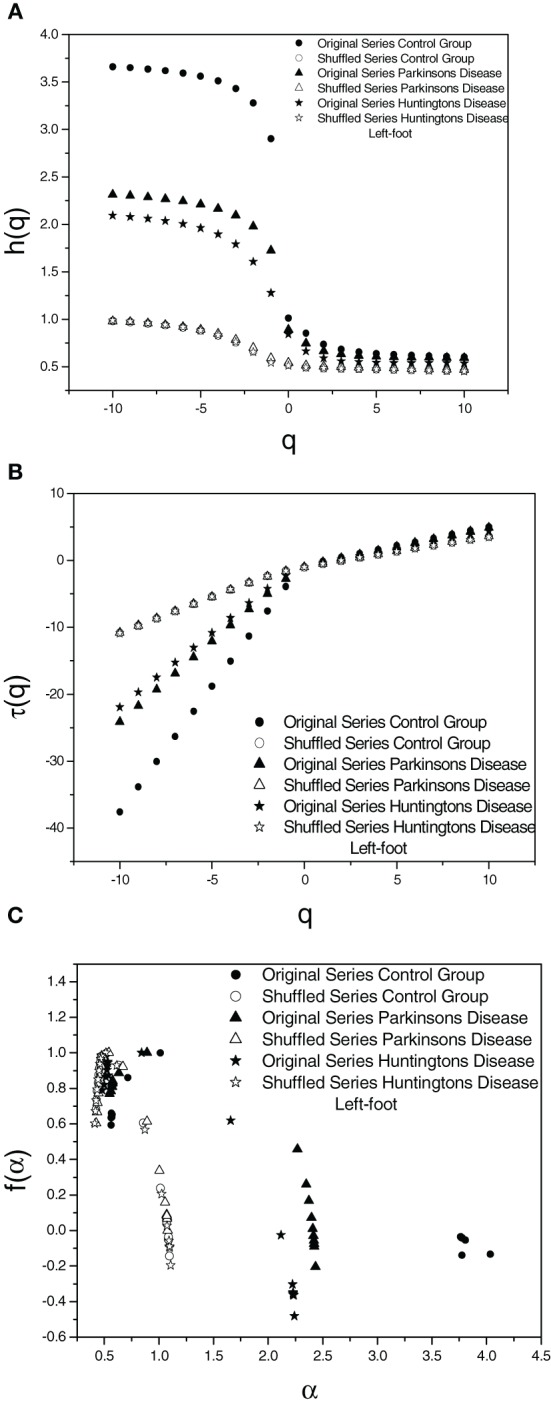
**Plot of (A) Generalized Hurst exponent *h*(*q*) vs. order *q* (B) Classical Scaling exponent (Equation 5) τ(*q*) vs. order *q* and (C) Dimension of subset series (Equation 6) *f*(α) vs. Singularity strength (Equation 7) α for original and shuffled series for one set each of three different cases for left-foot**.

To ascertain the origin of multifractality, the corresponding randomly shuffled series was analyzed for all the three cases. The variation of the values of *h*(*q*) vs. *q*, τ(*q*) vs. *q*, and *f*(α) vs. α, respectively, for the original series and the corresponding randomly shuffled series are represented in Figure 2 where the plots are shown for one subject for each group.

The values of *W*_shuffled_ and γ_shuffled_, the width of the multifractal spectra and correlation coefficient for the shuffled and original series for one subject in each group are shown in Table 2. A comparison of the values and figures suggests the fact that the origin of multifractality is due to both—broad probability distribution and long-range correlation, however, long-range correlation is dominant as suggested by the large reduction in multifractal width. Values of γ_shuffled_ are quite close to 1 when compared to lower values in the original series as expected since the correlations are destroyed in the shuffling process. This result demonstrates the fact that the multifractality in human gait is predominantly due to long-range correlations. The values of the autocorrelation coefficient reveal that the gait series is long range positive correlated series which approaches toward an uncorrelated series with neurodegenerative diseases.

**Table 2:** **Values of multifractal width *W* (Equation 9) and autocorrelation coefficient γ (Equation 10) for (i) healthy subjects (Control Group), (ii) subjects with Parkinson's disease, and (iii) subjects persons with Huntington's disease for both the original series and the shuffled series**.

		***W***_**original**_	***W***_**shuffled**_	γ_**original**_	γ_**shuffled**_
Control Group	left-foot	4.0 ± 0.1	0.93 ± 0.07	0.53	1.03
	right-foot	4.0 ± 0.2	1.2 ± 0.2	0.43	1.06
Parkinson's disease	left-foot	2.3 ± 0.2	0.88 ± 0.07	0.67	0.99
	right-foot	2.7 ± 0.1	0.93 ± 0.07	0.61	0.99
Huntington's disease	left-foot	2.20 ± 0.09	0.90 ± 0.08	0.82	1.03
	right-foot	2.07 ± 0.09	0.72 ± 0.05	0.86	1.07

## Conclusions

MFDFA was applied to analyze the stride-interval time-series obtained from the three groups (i) healthy persons (Control Group), (ii) persons with PD, and (iii) persons with HD, and the following facts have been revealed.

(i) The human gait rhythm exhibits multifractal properties, in all three cases. However, the multifractal properties are more pronounced in normal persons, i.e., degree of multifractality is greater in normal persons, than in persons with neurodegenerative diseases.

(ii) The left foot and the right foot data produces identical results in almost all the cases.

The MFDFA method is capable of distinguishing between normal and diseased set. ANOVA was employed to test the statistical significance of the data. The values of *F* and *p* are listed in Table 1. The values of *W* are found to different in normal and diseased set with a confidence level about 95%. However, when it comes to distinguishing between two diseased set the MFDFA method produces almost same results. Thus, it can be inferred that the neurodegenerative diseases can bring about an alteration in the fractal dynamics of human gait due to weakening and impairment of neural control on locomotion. The results are consistent with previous studies. Hausdorff et al. (1996, 1997, 2000, 2001, Goldberger et al., 2002b) have observed loss of correlation in inter-stride interval fluctuation with patients suffering from PD and HD. Scafetta et al. (2007) have observed that due to neuronal deterioration, a network of neurons controlling human gait is expected to be less correlated in diseased set than a healthy neuronal network. A leftward shift of the Hölder exponent distribution was observed and was estimated to increase with the severity of the neurodegenerative disease.

However, there are shortcomings of the MFDFA method which can lead to spurious results. Fractional Gaussian noise also mimics typical shape expected from long range correlations and hence can lead to erroneous results (Eke et al., 2012; Delignières and Marmelat, 2013). Bashan et al. (2008) have observed that MFDFA1 systematically overestimates the scale scaling exponents for small scale s. In this respect Centered Moving Average (CMA) (Alvarez-Ramirez et al., 2005) will be more fruitful in analyzing the scaling properties in short data sets without trends. However, for data with possible unknown trends application of standard DFA with several different detrending polynomial orders will help to distinguish between real crossovers and artificial crossovers due to trends.

### Conflict of interest statement

The authors declare that the research was conducted in the absence of any commercial or financial relationships that could be construed as a potential conflict of interest.
